# Gravity Modeling to Predict Patient Choice of Hospital for Pancreaticoduodenectomy

**DOI:** 10.1245/s10434-026-19450-2

**Published:** 2026-03-18

**Authors:** Katie Ross-Driscoll, Sarah Huber, Alexa J. Hughes, Kelly Yang, Adam S. Wilk, Karl Y. Bilimoria, Rachel E. Patzer, Ryan J. Ellis

**Affiliations:** 1 Department of Surgery, Indiana School of Medicine, Surgical Outcomes and Quality Improvement Center (SOQIC), Indianapolis, IN USA; 2 Division of Transplant, Department of Surgery, Indiana University School of Medicine, Indianapolis, IN USA; 3 Department of Business Economics and Public Policy, Kelley School of Business, Indiana University, Bloomington, IN USA; 4 William M. Tierney Center for Health Services Research, Regenstrief Institute, Indianapolis, IN USA; 5 Division of Surgical Oncology, Department of Surgery, Indiana University School of Medicine, Indianapolis, IN USA

## Abstract

**Background:**

Prior studies simulating how regionalization of complex surgeries such as pancreatoduodenectomy (PD) would affect access to care assumed that patients travel to their closest hospital. The validity of this assumption had not been evaluated. Our objectives were to assess the accuracy of a distance-only model in predicting where a patient received PD and determine whether incorporating hospital characteristics improved accuracy.

**Methods:**

Data on patients undergoing PD from 2016 to 2017 in New York or Florida were obtained from the Statewide Inpatient Databases. We assessed three models: distance alone (model 1), distance and hospital volume (model 2), and distance, hospital volume, rurality, cancer accreditation, medical school affiliation, and centralization taxonomy of the health system (model 3). Model accuracy was determined by comparing the predicted hospital and the actual hospital where the patient received care.

**Results:**

Among the 2949 included patients, model 1 was accurate for only 16.3%; model 2 was accurate for 32.9%, and model 3 was accurate for 24.3%. Patients who were accurately classified with models 2 or 3 but not 1 were more likely to live in major metropolitan areas (74.7% vs. 61.0%, *p* < 0.001), more likely to be non-Hispanic white (71.3% vs. 68.3%, *p* = 0.04), more likely to have private insurance (35.0% vs. 27.8%, *p* < 0.001), and had lower in-hospital mortality (1.3% vs. 3.3%, *p* = 0.03).

**Conclusions:**

Distance-only models were largely inaccurate for predicting where patients received PD. Simulations of patient redistribution should incorporate volume, distance, and additional patient- and provider-level factors.

Pancreaticoduodenectomy (PD) is among the most complex abdominal operations and associated with 1–2% perioperative mortality^1^ and complications affecting as many as 50% of patients.^2,3^ Oncologic and surgical outcomes after PD are superior when the operation is performed at a high-volume center (HVC) where specialized services and resources are concentrated.^4–13^ Although no formal policies exist mandating regionalization for PD in the United States, efforts are ongoing to determine ideal volume thresholds or other quality metrics by which to stratify centers.^13–17^

Though regionalization may lead to improved outcomes, centralization of surgical care risks exacerbating issues in access to care.^18,19^ Additionally, patients from racial and ethnic minority groups, uninsured patients, and patients from rural or low educational attainment areas are less likely to receive care for cancer at HVCs,^16,17,20–25^ and centralization may disproportionately impose greater travel burdens or logistical barriers to care on these groups. Many studies simulating centralization of PD focused on hospital-level or system-level implications;^14,26,27^ studies that did assess population-level impacts of PD regionalization assumed redistribution based on distance alone^28,29^ (i.e., simulated patients receiving care at the nearest center) without evaluating the accuracy of this approach.

However, although distance is an important factor in hospital choice, distance alone does not fully explain patient behavior.^30^ Other factors, including complex unmeasured phenomena such as hospital reputation and physician referral patterns, also play a significant role.^31^ In fact, most patients (84%) bypass their nearest center for PD,^32,33^ indicating that distance-only models of patient redistribution may not be sufficient. One alternative approach to simulating patient distribution is gravity modeling,^34^ a technique in spatial epidemiology in which measurable parameters affect the ‘attraction’ to each hospital and thus patient choices. We hypothesized that adding hospital parameters would significantly improve the accuracy of simulated patient choice in where to undergo surgery.

## Methods

### Data Sources and Study Population

Data were obtained from the Healthcare Cost and Utilization Project State Inpatient Databases from New York and Florida (2016–2017). Patients were included if they were adults aged >18 years who had an International Classification of Diseases and Related Health Problems, Tenth Revision code indicating pancreatoduodenectomy, specifically at least one of 0DB90ZX, 0DB90ZZ, 0DB94ZX, 0DB94ZZ, 0DT90ZZ, or 0DT94ZZ, in addition to at least one of 0FBG0ZX, 0FBG0ZZ, 0FBG4ZX, 0FBG4ZZ, 0FTG0ZZ, or 0FTG4ZZ^35^. Patients were excluded if they were missing a patient or hospital ZIP code (*n* = 63) or were from an out-of-state ZIP code (*n* = 577). Data on hospital characteristics were obtained from the 2016 American Hospital Association survey.

### Variables

Travel distance was calculated as the geodesic distance from the centroid of the patient’s home ZIP code to the centroid of the hospital’s ZIP code, in miles. Hospital volume was calculated by aggregating the number of patients with PD seen at that hospital in the cohort. Patient characteristics of interest included age, race (categorized as white vs. non-white), sex, rural-urban status (categorized as living in a major metropolitan area, a small metropolitan area, a micropolitan area, or a rural area), primary payer (categorized as Medicare, Medicaid, private, and other), and the social deprivation index (SDI) of the patient’s home ZIP code (higher SDI indicates greater degree of deprivation and lower socioeconomic status). Hospital characteristics included whether the hospital was recorded as having a medical school affiliation, the core-based statistical area designation of the hospital (grouped as metropolitan vs. micropolitan and rural), Commission on Cancer accreditation status, and the centralization taxonomy of the hospital as defined by the American Hospital Association survey (dichotomized as centralized health system, centralized physician/insurance health system, and moderately centralized health system [centralized] vs. decentralized health system and independent hospital system [not centralized]).

### Analysis

We compared three models to predict the attraction of patients (T_ij_) in ZIP code *i* to hospital *j*. Model 1, the distance-alone model, contained a parameter for the log of the distance between ZIP code *i* and hospital *j*. Model 2 contained a parameter for the log of the distance between ZIP code *i* and hospital *j* as well as the log of the volume of hospital *j*. Model 3 contained the same parameters as model 2, along with fixed effects for four hospital characteristics: medical school affiliation, rurality, cancer accreditation, and centralization. We applied a double-demeaning estimator using the gravity package in R to account for unobserved ZIP- and hospital-level factors; this approach minimizes between-hospital variation, meaning the resulting hospital fixed effects may improve prediction but should not be interpreted causally. We estimated model parameters jointly for the overall study population and then separately for each state.

For every patient (*k*), we identified all possible patient–hospital pairs (*k × j*). For each pair, we used the coefficients from the models described above to calculate a predicted probability. The *predicted* hospital was the one with the highest predicted probability for each patient. For hospitals with tied scores, the predicted hospital was selected randomly among the ties. The *observed* hospital was the one where the patient received surgery. Accuracy was calculated as the number of patients for whom the predicted and observed hospital agreed, divided by the total N. Accuracy was calculated for each model overall, and then separately for New York and Florida. We conducted a sensitivity analysis in which we defined accuracy as the observed hospital being in the top two or three predicted hospitals. We also evaluated accuracy among Medicare patients, whose insurance is presumably accepted by all hospitals. Travel distance for each model was calculated as the distance from the centroid of the top predicted hospital to the patient home ZIP.

We characterized patient subgroups, including patients who were accurately classified by model 1, patients who were accurately classified by models 2 or 3, and patients who were inaccurately classified by all models. In a sensitivity analysis, we examined in-hospital mortality among patients who were accurately versus inaccurately classified. All analyses were conducted using R version 4.5.0.

## Results

### Population

A total of 2949 patients who underwent PD in New York (*n* = 1417) and Florida (*n* = 1532) between 2016 and 2017 were included in the study. In Florida, patients received surgery at 72 hospitals, with a median volume of seven cases (interquartile range [IQR] 2.0–26.5). In New York, patients received surgery at 48 hospitals, with a median volume of 14.5 (IQR 3.0–32.5). The mean ± standard deviation (SD) age of patients at admission was 65.6 ± 12.3 years (Table 1). The majority of patients lived in a major metropolitan area (72.5%), were non-Hispanic white (65.5%), and had Medicare as their primary payer (55.0%). Patients in Florida were older (mean 66.3 vs. 64.8, *p* < 0.001), more likely to live in a small metropolitan area (35% vs. 10.9%, *p* < 0.001), more likely to be Hispanic (16.8% vs. 8.6%, p < 0.001), and less likely to be of other race or ethnicity (4.8% vs. 19.2%, *p* < 0.001) than patients in New York. Patients in New York were more likely to have Medicaid (15.2% vs. 5.6%, *p* < 0.001) or private insurance (35.6% vs. 26.7%, *p* < 0.001) and had higher SDI scores (51.6 vs. 48.9, *p* = 0.01) than patients in Florida.

**Table 1:** Demographic characteristics of patients undergoing pancreatoduodenectomy in New York and Florida, 2016–2017

Characteristic	Overall *N* = 2949	Florida *N* = 1532	New York *N* = 1417	*p*-value (FL vs. NY)
Age at admission, years	65.6 ± 12.3	66.3 ± 11.9	64.8 ± 12.8	<0.001
Sex				0.27
Male	1560 (52.9)	794 (51.8)	766 (54.1)	
Female	1385 (47.0)	734 (47.9)	651 (45.9)	
Rural-urban categorization				<0.001
Major metropolitan	2137 (72.5)	945 (61.7)	1192 (84.1)	
Small metropolitan	691 (23.4)	536 (35.0)	155 (10.9)	
Micropolitan or rural	121 (4.1)	51 (3.3)	70 (4.9)	
Race/ethnicity				<0.001
Non-Hispanic White	1931 (65.5)	1049 (68.5)	882 (62.2)	
Black	278 (9.4)	137 (8.9)	141 (10.0)	
Hispanic	380 (12.9)	258 (16.8)	122 (8.6)	
Other	346 (11.7)	74 (4.8)	272 (19.2)	
Payer				<0.001
Medicare	1623 (55.0)	957 (62.5)	666 (47.0)	
Medicaid	301 (10.2)	86 (5.6)	215 (15.2)	
Private	913 (31.0)	409 (26.7)	504 (35.6)	
Other	112 (3.8)	80 (5.2)	32 (2.3)	
SDI^a^	50.2 ± 29.3	48.9 ± 26.5	51.6 ± 32.1	0.01
Medical school-affiliated hospital	2081 (70.6)	772 (50.4)	1309 (92.4)	<0.001
Rural hospital	36 (1.2)	7 (0.5)	29 (2.0)	<0.001
CoC-accredited hospital	1975 (67.0)	1188 (77.5)	787 (55.5)	<0.001
Hospital in a centralized health system	1370 (46.5)	496 (32.4)	874 (61.7%)	<0.001

Data are presented as mean ± standard deviation or n (%) unless otherwise indicated

^a^SDI of patient’s home ZIP code (higher SDI indicates greater degree of deprivation and lower socioeconomic status)

CoC, Commission on Cancer; FL, Florida; NY, New York; SDI, Social Deprivation Index

Hospital characteristics varied across states. Most patients were treated at a hospital with a medical school affiliation (70.6%), but this was more common in New York (92.4%) than in Florida (50.4%, *p* < 0.001). However, more patients in Florida were treated at a Commission on Cancer-accredited hospital (77.5% vs. 55.5%, *p* < 0.001). More patients in New York were treated at a hospital in a centralized health system (61.7% vs. 32.4%, *p* < 0.001). Very few patients in either state were treated at a rural hospital (1.2%).

### Model Fit and Accuracy

Model 1 (distance alone) accurately classified 16.3% of patients (Table 2). Accuracy in the overall population was greater than with state-specific parameters (Florida: 15.0%; New York: 14.6%). Model 2 accurately classified 32.9% of patients, with 34.2% accuracy in Florida and 32.9% in New York. Overall, model 3 accurately classified 24.3% of patients. State-specific models improved accuracy, particularly in New York (Florida: 28.5%; New York: 33.9%). Over half of patients were not accurately classified by any of the three models, with the highest proportion inaccurately classified (57.1%) in New York. Accuracy was similar among only patients with Medicare (model 1: 16.0%; model 2: 34.0%; model 3: 23.7%).

**Table 2:** Accuracy of distance-alone model and gravity model in predicting observed location of care for patients who underwent pancreatoduodenectomy

	Accuracy of top predicted hospital^a^			Accuracy of top two predicted hospitals^b^			Accuracy of top three predicted hospitals^c^		
Model accuracy	Overall	Florida	New York	Overall	Florida	New York	Overall	Florida	New York
Model 1 sufficient	482 (16.3)	230 (15.0)	207 (14.6)	924 (31.3)	525 (34.3)	423 (29.9)	1194 (40.5)	651 (42.5)	537 (37.9)
Model 2 sufficient	970 (32.9)	524 (34.2)	466 (32.9)	1500 (50.9)	796 (52.0)	714 (50.4)	1939 (65.8)	1009 (65.9)	913 (64.4)
Model 3 sufficient	716 (24.3)	437 (28.5)	481 (33.9)	1159 (39.3)	617 (40.3)	680 (48.0)	1479 (50.2)	773 (50.5)	813 (57.4)
All models correct	191 (6.5)	92 (6.0)	119 (8.4)	499 (16.9)	287 (18.7)	295 (20.8)	766 (26.0)	422 (27.5)	447 (31.5)
No model correct	1512 (51.3)	813 (53.1)	809 (57.1)	1052 (35.7)	508 (33.2)	558 (39.4)	765 (25.9)	360 (23.5)	402 (28.4)

Data are presented as n (%)

^a^Accuracy of top predicted hospital is defined as when the observed hospital for a patient is the same as the hospital with the highest predicted probability from the model

^b^Accuracy of top two predicted hospitals is defined as when the observed hospital for a patient was among the two highest probability hospitals predicted by the model

^c^Accuracy of top three predicted hospitals is defined as when the observed hospital for a patient was among the three highest probability hospitals predicted by the model

Overall, model 1 was accurate in identifying the observed hospital in the top two predicted hospitals 31.3% of the time, model 2 was accurate 50.9% of the time, and model 3 was accurate 39.3% of the time (Table 2). No model identified the observed hospital in the top two predicted hospitals for 1052 patients (35.7%). When considering whether the observed hospital was in the top three predicted hospitals, only 25.9% of patients were inaccurately classified by all models. Models 1 and 2 had better accuracy in Florida than in New York for the top two and top three predicted hospitals, and model 3 had better accuracy in New York. Model 3 was less accurate in the pooled overall population than in either state separately.

Relative to observed travel distance, model 1 underestimated predicted travel distance by 15.1 ± 35.1 miles. Model 2 underestimated travel distance by 8.3 ± 39.0 miles, and model 3 underestimated travel distance by 10.1 ± 36.1 miles. In Florida, model 1 underestimated travel distance relative to observed by 20.2 ± 43.2 miles, model 2 underestimated travel distance by 12.5 ± 43.5 miles, and model 3 underestimated travel distance by 14.2 ± 43.3 miles. In New York, model 1 underestimated travel distance relative to observed by 9.6 ± 22.2 miles, model 2 underestimated travel distance relative to observed by 4.1 ± 25.8 miles, and model 3 underestimated travel distance by 6.1 ± 21.7 miles.

### Patient Subgroups

Patients accurately classified by models 2 or 3 but not model 1 were more likely to live in major metropolitan areas (74.7% vs. 61.0%, *p* < 0.001), more likely to be non-Hispanic white (71.3% vs. 68.3%, *p* = 0.04) and more likely to have private insurance (35.0% vs. 27.8%, *p* < 0.001) than patients accurately classified by model 1 (Table 3). In Florida, patients accurately classified by models 2 or 3 but not model 1 were less likely to be Black (6.5% vs. 12.2%) and more likely to be Hispanic (18.8% vs. 14.8%, *p* = 0.01) than patients accurately classified by model 1. In New York, patients accurately classified by models 2 or 3 but not model 1 were more likely to live in a major metropolitan area (79.1% vs. 69.6%, *p* = 0.03) and less likely to live in disadvantaged neighborhoods (mean SDI 43.2 vs. 50.0, *p* < 0.001) than patients accurately classified by model 1.

**Table 3:** Characteristics of patients undergoing pancreatoduodenectomy who were accurately classified by the distance-alone model compared with those who were accurately classified by only the gravity model, overall and stratified by state (2016–2017)^a^

	Overall			Florida			New York		
Characteristic	Model 1 (*n* = 482)	Model 2 or 3 (*n* = 955)	*p*-value	Model 1 (*n* = 230)	Model 2 or 3 (*n* = 489)	*p*-value	Model 1 (*n* = 207)	Model 2 or 3 (*n* = 401)	*p*-value
Age at admission, years	66.0 ± 12.8	65.9 ± 12.2	0.89	66.9 ± 13.0	67.2 ± 11.0	0.78	65.5 ± 11.4	66.0 ± 11.8	0.64
Sex			0.82			0.88			0.32
Male	258 (53.5)	503 (52.7)		119 (51.7)	257 (52.6)		119 (57.5)	212 (52.9)	
Female	224 (46.5)	451 (47.2)		111 (48.3)	231 (47.2)		88 (42.5)	189 (47.1)	
Rural–urban categorization			<0.001			0.11			0.03
Major metropolitan	294 (61.0)	713 (74.7)		116 (50.4)	285 (58.3)		144 (69.6)	317 (79.1)	
Small metropolitan	163 (33.8)	199 (20.8)		103 (44.8)	189 (38.7)		43 (20.8)	60 (15.0)	
Micropolitan or rural	25 (5.2)	43 (4.5)		11 (4.8)	15 (3.1)		20 (9.7)	24 (6.0)	
Race/ethnicity			0.04			0.01			0.09
Non-Hispanic White	329 (68.3)	681 (71.3)		163 (70.9)	340 (69.5)		151 (72.9)	323 (80.5)	
Black	48 (10.0)	62 (6.5)		28 (12.2)	32 (6.5)		20 (9.7)	25 (6.2)	
Hispanic	63 (13.1)	106 (11.1)		34 (14.8)	92 (18.8)		17 (8.2)	18 (4.5)	
Other	39 (8.1)	101 (10.6)		3 (1.3)	21 (4.3)		19 (9.2)	35 (8.7)	
Payer			<0.001			0.16			0.16
Medicare	269 (55.8)	528 (55.3)		149 (64.8)	310 (63.4)		98 (47.3)	192 (47.9)	
Medicaid	62 (12.9)	60 (6.3)		20 (8.7)	26 (5.3)		25 (12.1)	35 (8.7)	
Private	134 (27.8)	334 (35.0)		49 (21.3)	132 (27.0)		75 (36.2)	166 (41.4)	
Other	17 (3.5)	33 (3.5)		12 (5.2)	21 (4.3)		9 (4.3)	8 (2.0)	
SDI^b^	48.7 ± 29.1	46.1 ± 29.8	0.12	50.1 ± 27.2	47.0 ± 26.1	0.15	50.0 ± 29.0	42.3 ± 30.6	<0.001

Data are presented as mean ± standard deviation or n (%) unless otherwise indicated

^a^Characteristics of patients who were accurately classified by the distance-alone model or accurately by both the distance-alone model and the gravity model compared with patients who were only accurately classified by the gravity model. Patients who were not classified accurately by either model were excluded

^b^SDI of patient’s home ZIP code (higher SDI indicates greater degree of deprivation and lower socioeconomic status)

SDI, social deprivation index

Compared with patients who were accurately classified by at least one model, patients who were inaccurately classified by all models were more likely to live in major metropolitan areas (74.7% vs. 70.1%, *p* = 0.01), less likely to be non-Hispanic white (60.9% vs. 70.3%, *p* < 0.001), less likely to have private insurance (29.4% vs. 32.6%, *p* = 0.01), and lived in areas with higher neighborhood socioeconomic deprivation (mean SDI 53.3 vs. 47.0, *p* < 0.001). This pattern was similar in New York; in Florida, only rural-urban categorization was associated with model accuracy (Table 4).

**Table 4:** Characteristics of patients undergoing pancreatoduodenectomy who were accurately classified by at least one model compared with those who were inaccurately classified by all models, overall and stratified by state (2016–2017)

	Overall			Florida			New York		
Characteristic	No model accurate (*n* = 1512)	Any model accurate (*n* = 1437)	*p*-value	No model accurate (*n* = 813)	Any model accurate (*n* = 719)	*p*-value	No model accurate (*n* = 809)	Any model accurate (*n* = 608)	*p*-value
Age at admission, years	65.3 ± 12.3	65.9 ± 12.4	0.15	65.6 ± 12.1	67.1 ± 11.7	0.02	64.0 ± 13.5	65.8 ± 11.7	0.01
Sex			0.99			0.81			0.84
Male	799 (53.0)	761 (53.0)		418 (51.4)	376 (52.3)		435 (53.8)	331 (54.4)	
Female	710 (47.0)	675 (47.0)		392 (48.2)	342 (47.6)		374 (46.2)	277 (45.6)	
Rural–urban categorization			0.01			<0.01			<0.01
Major metropolitan	1130 (74.7)	1007 (70.1)		544 (66.9)	401 (55.8)		731 (90.4)	461 (75.8)	
Small metropolitan	329 (21.8)	362 (25.2)		244 (30.0)	292 (40.6)		52 (6.4)	103 (16.9)	
Micropolitan or rural	53 (3.5)	68 (4.7)		25 (3.1)	26 (3.6)		26 (3.2)	44 (7.2)	
Race/ethnicity			<0.001			0.06			<0.01
Non-Hispanic White	921 (60.9)	1010 (70.3)		546 (67.2)	503 (70.0)		408 (50.4)	474 (78.0)	
Black	168 (11.1)	110 (7.7)		77 (9.5)	60 (8.3)		96 (11.9)	45 (7.4)	
Hispanic	211 (14.0)	169 (11.8)		132 (16.2)	126 (17.5)		87 (10.8)	35 (5.8)	
Other	206 (13.6)	140 (9.7)		50 (6.2)	24 (3.3)		218 (26.9)	54 (8.9)	
Payer			0.01			0.25			<0.01
Medicare	826 (54.6)	797 (55.3)		498 (61.3)	459 (63.8)		376 (46.5)	290 (47.7)	
Medicaid	179 (11.8)	122 (8.5)		40 (4.9)	46 (6.4)		155 (19.2)	60 (9.9)	
Private	445 (29.4)	468 (32.6)		228 (28.0)	181 (25.2)		263 (32.5)	241 (39.6)	
Other	62 (4.1)	50 (3.5)		47 (5.8)	33 (4.6)		15 (1.9)	17 (2.8)	
SDI^a^	53.3 ± 28.8	47.0 ± 29.6	<0.001	49.8 ± 26.4	47.9 ± 26.5	0.17	56.7 ± 32.6	45.0 ± 30.2	<0.01

Data are presented as mean ± standard deviation or n (%) unless otherwise indicated

^a^SDI = social deprivation index of patient’s home ZIP code (higher SDI indicates greater degree of deprivation and lower socioeconomic status)

SDI, Social Deprivation Index

In-hospital mortality was 2.0% among patients who were accurately classified by at least one model, compared with 4.1% among patients who were inaccurately classified by all models (*p* = 0.002). In-hospital mortality was 1.3% for patients who were accurately classified by models 2 or 3 but not model 1 and 3.3% among patients who were accurately classified by model 1 (*p* = 0.03).

## Discussion

Our study demonstrates that gravity modeling that includes hospital parameters more accurately predicts where patients undergo PD than does distance alone, though overall accuracy remained low. Patients for whom accuracy improved by including hospital characteristics were more likely to live in major metropolitan areas, be non-Hispanic white, and have private insurance. Patients inaccurately classified by all models were more also likely to live in major metropolitan areas but less likely to be non-Hispanic white or have private insurance. They also lived in neighborhoods with more socioeconomic deprivation. Adding volume alone improved accuracy more than adding additional hospital characteristics, particularly in the overall population, highlighting the complexity of hospital choice dynamics. All models underestimated actual travel, indicating that inaccurate models of hospital choice may misrepresent the true potential burden of surgical centralization in previously published simulations.

Previous studies simulating redistribution of patients undergoing PD to HVCs reallocated patients to optimize postoperative quality metrics,^14^ minimize healthcare costs,^26^ or determine the optimal location-allocation market for pancreatic resections.^27^ These studies showed minimal impacts on travel burden for patients under the assumption that patients would be treated at the nearest facility.^28,29^ Our results challenge the validity of this assumption: the distance-alone model had poor accuracy for predicting patient hospital choice. This is consistent with literature showing that more than 80% of patients undergoing PD bypassed their closest facility,^32,33,36^ and one in five patients who underwent PD at a low-volume center bypassed an HVC.^32^ More recent data on other complex surgeries suggest that travel time for rural patients has nearly doubled in the last 15 years,^37^ further highlighting how prior models may have inaccurately predicted the effects of centralization.

Accurate policy simulation is critical to avoid unintentionally exacerbating disparities in access to surgical care. Patients from disadvantaged areas are over 70% more likely to bypass an HVC and receive care at a low-volume center,^32^ and in our analysis these patients were more likely to be misclassified by all models. One reason for this inaccurate classification may be that these patients have less power to “choose” where they receive care based on hospital characteristics. We also observed higher in-hospital mortality among patients who were inaccurately classified by all models and among patients for whom model 1 was sufficient, indicating that these patients also have worse surgical outcomes. Policies built on such models will likely misclassify these patients and therefore risk widening existing disparities in access to high-quality care.

Geographic heterogeneity further complicates prediction. Our findings suggest that distance-alone models underestimated true travel burden by approximately 15 miles per patient; the felt impact of these 15 miles on patients is likely to vary strongly by geographic context (i.e. traffic density, availability of public transportation). We also observed state-specific heterogeneity in model performance, with superior accuracy of model 3 in New York compared with in Florida. These findings highlight that healthcare delivery is strongly influenced by local contextual factors, in addition to broader secular trends such as the increasing push for centralization. The impact of these trends may be felt differently across geographic regions.

None of the models performed well in predicting where patients ultimately received care. Importantly, adding hospital characteristics beyond volume did not meaningfully improve model performance, particularly in the overall population. This analysis focused on a limited set of hospital characteristics; however, hospital choice is likely influenced by a broader range of considerations, including physician referral patterns, surgeon interpersonal skills, ease of parking, publicly available quality reports, continuity of care, and insurance coverage terms and provider networks,^38–42^ among others. Hospital choice dynamics are complex and likely vary by context – factors that attract patients in one market may have little influence in another. Patients are also heterogeneous in how they weigh surgical risk, hospital quality, and travel distance^30,38,43–46^ when making decisions about where to seek care. Our approach models aggregate patient flows rather than individual-level decision-making; future work using more complex modeling approaches may be useful in identifying interactions between patient and hospital characteristics that influence hospital choice. Accurate models are key to performing adequate simulation of patient redistribution in response to both formal policy and secular trends (e.g. health systems consolidation,^47^ rural hospital closures,^5^ and reimbursement structure change).

This analysis has important limitations. First, our data are from 2016–2017, and factors influencing hospital choice may have changed over time. However, we believe that our main finding – that distance alone is not sufficient to predict where patients receive care – is unlikely to have changed. Second, although the State Inpatient Database captures state admissions data longitudinally and includes all-payer sources, we were unable to capture out-of-state hospitals. As a result, we did not capture care received at nationally recognized “destination hospitals”, to which some patients travel substantial distances; consequently, observed travel distances are likely underestimated. Travel distance was estimated using the centroid of both the patient and the hospital ZIP code and may be more accurately estimated using address or drive time data. Third, we excluded out-of-state patients, who would have falsely inflated accuracy model accuracy as their home state hospitals are not included. This may limit the generalizability of the model and potentially underestimate hospital volume. More regional or national data, particularly of contiguous states, would improve the understanding of geographic patterns of hospital choice. Fourth, information about health systems within the state is not included in the State Inpatient Databases and could significantly improve the accuracy of the model by accounting for intra-system referrals. Finally, we calculated volume as the number of surgeries performed during the study period, but volume from the prior year would more accurately reflect the information available to patients at the time of hospital choice.

## Conclusion

Gravity models that incorporate hospital characteristics outperform distance-only models in predicting patient hospital choice for PD, but their accuracy remains limited. Model performance varies by geography and patient characteristics, with disadvantaged patients particularly vulnerable to misclassification. Policy simulation relying on distance-alone models risks underestimating the burden of regionalization, potentially exacerbating disparities in access to high-quality surgical care. More accurate models incorporating a broader set of hospital, patient, and market-level factors are needed to better estimate the population-level impact of centralization.
